# Availability and Costs of Allergic Rhinitis Treatments Across the World: A Survey of ARIA Experts

**DOI:** 10.1111/all.70340

**Published:** 2026-04-18

**Authors:** Maria Clara Urbano, Rafael José Vieira, Yi‐Kui Xiang, Sara Gil‐Mata, Anna Bedbrook, Baharudin Abdullah, Mona Al‐Ahmad, Maryam Ali Al‐Nesf, Emilio Alvarez‐Cuesta, Salma Mussagy Amade, Julijana Asllani, Kazi S. Bennoor, Elena Camelia Berghea, Mario Calvo‐Gil, Luis R. Caraballo, Elizabeth Castro, Lorenzo Cecchi, Deepa Choudhury, George Christoff, Derek K. Chu, Ieva Cirule, Alvaro A. Cruz, Dejan Dokic, Bilun Gemicioglu, Maria Antonieta Guzman, Ineta Grisle, Tari Haahtela, Elham Hossny, Wolfram Hoetzenecker, Martin Hrubisko, Tomohisa Iinuma, Zhanat Ispayeva, Juan Carlos Ivancevich, Fanny Wai San Ko, Helga Kraxner, Maciej Kupczyk, Violeta Kvedariene, Yen Lam, Désirée E. Larenas‐Linnemann, Bassam Mahboub, Padukudru A. Mahesh, Mohammed Reza Masjedi, Garry McDonald, Amarilis Melendez, Florin Mihaltan, Branislava Milenkovic, Yousser Mohammad, Marek Niedoszytko, Robyn E. O'Hehir, Carmen Panaitescu, Petr Panzner, Nikolaos G. Papadopoulos, José Miguel Fuentes Perez, Nhân Pham‐Thi, Constantinos Pitsios, Davor Plavec, María Susana Repka‐Ramirez, Jan Romantowski, Marylin Valentin Rostan, Menachem Rottem, Hani Salim, Pongsakorn Tantilipikorn, Vesna Tomic‐Spiric, Arunas Valiulis, Leticia de las Vecillas, Maria Teresa Ventura, Dana Wallace, Pascal Werminghaus, Sian Williams, Francisco Javier Plaza Zamora, Ludger Klimek, Torsten Zuberbier, Ana Margarida Pereira, João A. Fonseca, Jean Bousquet, Bernardo Sousa‐Pinto

**Affiliations:** ^1^ MEDCIDS—Department of Community Medicine, Information and Health Decision Sciences, Faculty of Medicine University of Porto Porto Portugal; ^2^ CINTESIS@RISE ‐ Centre for Health Technology and Services Research, Health Research Network, Faculty of Medicine University of Porto Porto Portugal; ^3^ Institute of Allergology Charité—Universitätsmedizin Berlin, Corporate Member of Freie Universität Berlin and Humboldt‐Universität Zu Berlin Berlin Germany; ^4^ Fraunhofer Institute for Translational Medicine and Pharmacology ITMP Immunology and Allergology Berlin Germany; ^5^ Shanghai Skin Disease Hospital, Tongji University School of Medicine Shanghai China; ^6^ ARIA (Allergic Rhinitis and Its Impact on Asthma) Montpellier France; ^7^ Department of Otorhinolaryngology, Head and Neck Surgery, School of Medical Sciences Universiti Sains Malaysia Kubang Kerian Kelantan Malaysia; ^8^ Microbiology Department College of Medicine, Kuwait University Kuwait City Kuwait; ^9^ Adult Allergy and Immunology Division Hamad Medical Corporation Doha Qatar; ^10^ Academic in Allergy and Immunology, Allergy Division Former Head, Ramon & Cajal University Hospital Madrid Spain; ^11^ Department of Internal Medicine Maputo Central Hospital Maputo Mozambique; ^12^ Department of Internal Medicine University of Medicine Tirana Albania; ^13^ Department of Respiratory Medicine National Institute of Diseases of the Chest and Hospital Dhaka Bangladesh; ^14^ Department of Pediatrics Carol Davila University of Medicine and Pharmacy Bucharest Romania; ^15^ Marie Curie Emergency Children's Hospital Bucharest Romania; ^16^ Pediatrics Department Universidad Austral de Chile Valdivia Chile; ^17^ Institute for Immunological Research University of Cartagena, Campus de Zaragocilla Cartagena de Indias Colombia; ^18^ Allergy and Immunology, Faculty of Medicine University of Buenos Aires Buenos Aires Argentina; ^19^ Allergy and Clinical Immunology Unit San Giovanni di Dio Hospital Florence Italy; ^20^ Paediatric Allergy Clinic, Department of Dermatology Amersham Hospital‐NHS Hospital Trust Amersham UK; ^21^ Bulgarian Alliance for Clinical and Translational Allergy Sofia Bulgaria; ^22^ Department of Health Research Methods, Evidence, and Impact & Department of Medicine McMaster University Hamilton Ontario Canada; ^23^ Evidence in Allergy Group McMaster University and the Research Institute of St. Joe's Hamilton Hamilton Ontario Canada; ^24^ Latvian Association of Allergists University Children Hospital Riga Latvia; ^25^ Fundacao ProAR and (Faculdade de Medicina da) Universidade Federal da Bahia Salvador Bahia Brazil; ^26^ Medical Faculty Skopje University Clinic of Pulmology and Allergy Skopje North Macedonia; ^27^ Department of Pulmonary Diseases, Cerrahpaşa Faculty of Medicine Istanbul University‐Cerrahpaşa Istanbul Turkey; ^28^ Department of Pulmonary Diseases, Institute of Pulmonology and Tuberculosis Istanbul University‐Cerrahpaşa Istanbul Turkey; ^29^ Immunology and Allergy Division Clinical Hospital, University of Chile Santiago Chile; ^30^ Riga East University Hospital Riga Latvia; ^31^ Skin and Allergy Hospital, Helsinki University Hospital, and University of Helsinki Helsinki Finland; ^32^ Pediatric Allergy, Immunology and Rheumatology Unit Children's Hospital, Ain Shams University Cairo Egypt; ^33^ Department of Dermatology and Venerology, Medical Faculty Johannes Kepler University Linz Austria; ^34^ Clinical Research Institute for Inflammation Medicine, Medical Faculty Johannes Kepler University Linz Austria; ^35^ Department of Allergy and Clinical Immunology St. Elisabeth's Oncology Institute Bratislava Slovakia; ^36^ Department of Otorhinolaryngology Chiba University Chiba Japan; ^37^ Department of Allergology and Clinical Immunology, Kazakhstan Association of Allergology and Clinical Immunology Kazakh National Medical University Almaty Kazakhstan; ^38^ Servicio de Alergia e Immunologia Clinica Santa Isabel Buenos Aires Argentina; ^39^ Department of Medicine and Therapeutics The Chinese University of Hong Kong Hong Kong China; ^40^ Department of Otorhinolaryngology, Head and Neck Surgery Semmelweis University Budapest Hungary; ^41^ Division of Internal Medicine, Asthma and Allergy Barlicki University Hospital, Medical University of Lodz Lodz Poland; ^42^ Institute of Clinical Medicine, Clinic of Chest Diseases and Allergology, Faculty of Medicine Vilnius University Vilnius Lithuania; ^43^ Institute of Biomedical Sciences, Department of Pathology, Faculty of Medicine Vilnius University Vilnius Lithuania; ^44^ People's Hospital 115, University Hospital Ho Chi Minh City Vietnam; ^45^ Center of Excellence in Asthma and Allergy Médica Sur Clinical Foundation and Hospital México City Mexico; ^46^ Pulmonary Department Rashid Hospital, DUBAI Health Dubai UAE; ^47^ Research Institute of Medical and Health Sciences University of Sharjah Sharjah UAE; ^48^ Department of Respiratory Medicine JSS Medical College, JSS Academy of Higher Education and Research Mysuru India; ^49^ Tobacco Control Research Center (TCRC), Iranian Anti‐Tobacco Association Tehran Iran; ^50^ Department of Pulmonary Medicine, School of Medicine Shahid Beheshti University of Medical Sciences Tehran Iran; ^51^ International Primary Care Respiratory Group IPCRG Edinburgh UK; ^52^ Chief Department of Otorhinolaryngology Hospital Santo Tomas Panama Panama; ^53^ UMF‐University of Medicine and Pharmacy ‘Carol Davila’, Pneumology Department National Institute of Pneumology ‘Marius Nasta’ Bucharest Romania; ^54^ Clinic for Pulmonary Diseases, Clinical Center of Serbia, Faculty of Medicine University of Belgrade, Serbian Association for Asthma and COPD Belgrade Serbia; ^55^ National Center for Research in Chronic Respiratory Diseases Collaborating with WHO—EMRO Tishreen University School of Medicine Latakia Syria; ^56^ Pharmacy Department Al‐Sham Private University Damascus Syria; ^57^ Department of Allergology Medical University of Gdansk Gdansk Poland; ^58^ Allergy, Asthma and Clinical Immunology, Alfred Health, Department of Immunology, School of Translational Medicine Monash University Melbourne Victoria Australia; ^59^ Center of Immuno‐Physiology and Biotechnologies, Department of Functional Sciences ‘Victor Babes’ University of Medicine and Pharmacy Timisoara Romania; ^60^ Timis County Emergency Clinical Hospital “Pius Brinzeu” Timisoara Romania; ^61^ Department of Immunology and Allergology, Faculty of Medicine in Pilsen Charles University Prague Czech Republic; ^62^ Allergy Department 2nd Pediatric Clinic, University of Athens Athens Greece; ^63^ Private Practice Mexico City Mexico; ^64^ Ecole Polytechnique de Palaiseau Palaiseau France; ^65^ IRBA (Institut de Recherche Bio‐Médicale Des Armées) Brétigny sur Orge France; ^66^ Université Paris Cité Paris France; ^67^ Center for Allergy and Clinical Immunology Alésia Paris France; ^68^ Medical School University of Cyprus Nicosia Cyprus; ^69^ Faculty of Medicine J.J. Strossmayer University of Osijek Osijek Croatia; ^70^ Prima Nova Zagreb Croatia; ^71^ Department of Allergy Clinics Hospital, National University San Lorenzo Paraguay; ^72^ Pediatrics, Allergy & Immunology, Latín American Society of Allergy Asthma & Immunology (SLAAi) Montevideo Uruguay; ^73^ Division of Allergy, Asthma and Clinical Immunology Emek Medical Center Afula Israel; ^74^ Rappaport Faculty of Medicine Technion‐Israel Institute of Technology Haifa Israel; ^75^ Department of Family Medicine, Faculty of Medicine and Health Sciences Universiti Putra Malaysia Selangor Malaysia; ^76^ Center of Research Excellence in Allergy & Immunology, Department of Otolaryngology, Faculty of Medicine Siriraj Hospital Mahidol University Bangkok Thailand; ^77^ Clinic of Allergology and Immunology University Clinical Center of Serbia Belgrade Serbia; ^78^ Faculty of Medicine University of Belgrade Belgrade Serbia; ^79^ Institute of Clinical Medicine and Institute of Health Sciences Medical Faculty of Vilnius University Vilnius Lithuania; ^80^ Clinic of Asthma Allergy and Chronic Lung Diseases Vilnius Lithuania; ^81^ Department of Allergy Hospital La Paz Institute for Health Research (IdiPAZ) Madrid Spain; ^82^ University of Bari Medical School Bari Italy; ^83^ Institute of Sciences of Food Production National Research Council (ISPA‐CNR) Bari Italy; ^84^ Nova Southeastern University College of Allopathic Medicine Fort Lauderdale Florida USA; ^85^ ENT and Allergology Düsseldorf Germany; ^86^ Sefac (Spanish Society of Clinical, Family and Community Pharmacy) Madrid Spain; ^87^ Department of Otolaryngology, Head & Neck Surgery Universitätsmedizin Mainz Mainz Germany; ^88^ Center for Rhinology and Allergology Wiesbaden Germany; ^89^ Allergy Unit Instituto and Hospital CUF Porto Portugal

**Keywords:** allergic rhinitis, health economics, intranasal corticosteroids, oral antihistamines, survey

## Abstract

**Introduction:**

Medication availability and costs can be highly variable across countries and in different time periods. We aimed to conduct a survey among local experts to obtain information on the availability and costs of allergic rhinitis medications in different countries.

**Methods:**

We sent a survey to members of the Allergic Rhinitis and its Impact on Asthma (ARIA) group, asking for the availability and the lowest cost of specific allergic rhinitis medications in their respective countries. Data in local currencies were converted to 2024 US Dollars adjusted for Purchasing Power Parity (PPP). We compared the costs of different medication classes, assuming, for each class, the least expensive drug in each country, as well as full treatment adherence.

**Results:**

We received responses from ARIA experts in 51 different countries. Intranasal corticosteroids (INCS) and oral antihistamines (OAH) were available in all countries, but this was not observed for intranasal antihistamines (INAH) or for INAH+INCS. In most countries, OAH was the least costly drug class, while INAH+INCS was the most expensive. Among INCS, beclomethasone was the medication most frequently identified as the cheapest, while cetirizine and loratadine were the OAH most frequently reported as the least expensive. Among INAH+INCS, azelastine‐fluticasone was more frequently identified as less costly than olopatadine‐mometasone.

**Conclusion:**

There is an important across‐country and across‐class variability in terms of costs of allergic rhinitis medications. The results of this study will inform the ARIA‐EAACI 2024–2025 guidelines.

## Graphical abstract

- We surveyed experts of the ARIA group on the costs of medications for allergic rhinitis in the respective countries.
- Overall, oral antihistamines are the cheapest medications while fixed combinations of antihistamines + corticosteroids are the most expensive.
- Overall, the lowest medication costs were found in Asia, while the highest were reported in South America.

Abbreviations: ARIA, Allergic Rhinitis and its Impact on Asthma; INCS, intranasal corticosteroids; INAH, intranasal antihistamines; OAH, oral antihistamines; USD PPP, US dollars adjusted for Purchasing Power Parity.

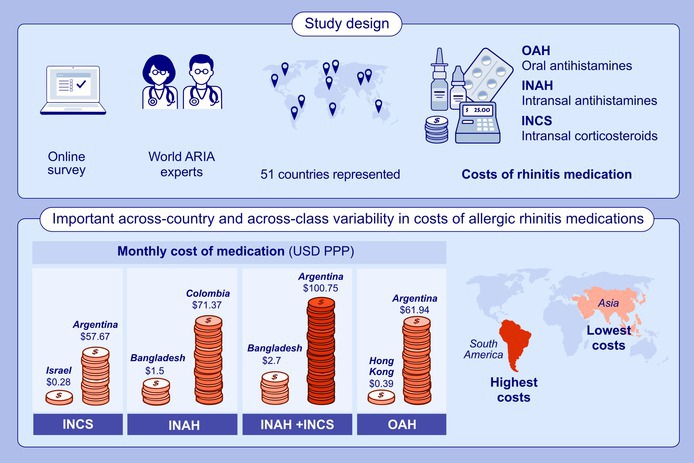

## 1 Introduction

Several medication classes can be used in the treatment of allergic rhinitis (AR), including, among others, intranasal corticosteroids (INCS), intranasal antihistamines (INAH), fixed combinations of INAH+INCS, and oral antihistamines (OAH). These medication classes differ not only in their efficacy and safety [1, 2] but also in other aspects such as acceptability, impact on equity, or costs. All these aspects should be considered when drafting recommendations on AR treatment. In fact, the Evidence‐to‐Decision (EtD) framework considers 12 criteria for which information is needed to inform guideline recommendations [3, 4]. Among them, three deal with resources required (costs) and cost‐effectiveness.

The importance of considering information on costs for decision‐making should not be downplayed: less affordable treatments may not be accessible to all patients, which can lead to important consequences in terms of equity. Affordability of the treatments may also have an impact on adherence. In addition, information on costs is necessary to identify which medications are cost‐effective. However, obtaining such information can be challenging, because there is a high across‐country variability in medication costs (and availability) and the costs of medications also tend to change with time. For example, the introduction of generic drugs into the market creates price competition, resulting in a substantial decrease in the price of medications. This process, however, varies between products and countries, which highlights the relevance of obtaining data on costs for each country [5]. Private companies often have data on medication costs, but geographical coverage is frequently limited.

The geographical differences in medication costs may also result in variations on which individual medications are the cheapest or on medication classes across different countries. Therefore, to inform the decision‐making process, evidence on the costs of rhinitis medications should be both current and geographically diverse. Unfortunately, such comprehensive and updated evidence is lacking, with most published studies being over 10 years old and restricted to specific countries [6, 7, 8, 9, 10, 11].

Therefore, and in the context of the development of the 2024–2025 update of the Allergic Rhinitis and its Impact on Asthma (ARIA) – European Academy of Allergy and Clinical Immunology (EAACI) Guidelines (ARIA‐EAACI 2024–2025) [12, 13], we aimed to conduct a survey among local experts to obtain estimates of the costs of AR medications across the different countries of the world.

## 2 Methods

### 2.1 Study Design

We conducted a cross‐sectional study to assess the availability and costs of different pharmacological treatments for AR across countries in 2024. In particular, we sent a survey to experts of the ARIA group, questioning them on the availability and lowest cost of several individual rhinitis medications (from different drug classes) in their respective countries. Our selection of ARIA experts was chosen for convenience: we needed to be sure that they understood our briefing and purpose. In addition, they were likely to have efficient networks in order to find data that were not necessarily publicly available. We encouraged the ARIA experts to forward the survey to other colleagues in countries where data were absent.

### 2.2 Participants

To be eligible for this study, the participants had to be members of the ARIA group or health professionals indicated by ARIA experts. The ARIA group is a non‐governmental organization dedicated to educating and implementing evidence‐based management for AR and asthma worldwide [14].

### 2.3 Data Collection

We created a survey asking ARIA members about the availability (either on prescription or over‐the‐counter) and lowest costs of a set of medications commonly used to treat AR (Table 1). In particular, we asked for the availability and costs of INCS, INAH, INAH+INCS, oral antihistamines (OAH), alongside other nasal, oral, and ocular medications for rhinitis.

**TABLE 1:** Allergic rhinitis medications for which costs were evaluated.

Drug class	Drugs
Intranasal corticosteroids	Beclomethasone dipropionate
	Budesonide
	Ciclesonide
	Dexamethasone
	Flunisolide
	Fluticasone furoate
	Fluticasone propionate
	Mometasone furoate
	Triamcinolone acetonide
Intranasal antihistamines	Azelastine 0.10%
	Azelastine 0.15%
	Levocabastine
	Olopatadine
Fixed combinations of intranasal antihistamines and corticosteroids	Azelastine‐fluticasone
	Olopatadine‐mometasone
Oral antihistamines	Bilastine
	Cetirizine
	Desloratadine
	Ebastine
	Fexofenadine
	Levocetirizine
	Loratadine
	Rupatadine

In terms of costs, participants were requested to report the current lowest cost available in their country for each treatment, considering the monthly cost per patient and assuming full adherence to the prescribed treatment. Participants were asked to present costs in their local currency. Of note, for each medication, the survey asked whether a generic was available.

The survey was sent to all ARIA experts by email in July 2024, with a reminder in the three subsequent months. In addition, the survey was publicized on ARIA group webinars and in meetings presenting scientific results of the ARIA group.

### 2.4 Data Analysis

Categorical variables were described using absolute and relative frequencies, while continuous variables were described using ranges.

We converted cost estimates provided in local currencies into 2024 US Dollars adjusted for Purchasing Power Parity (USD PPP) (https://data.worldbank.org/indicator/PA.NUS.PPP). This enabled estimates from different countries to be compared by adjusting them to the respective purchasing powers.

In cases where there was more than one answer per country, with different provided costs for the same drug, we considered the average value if the difference was lower than 5 USD PPP. If the difference was higher than 5 USD PPP, a third specialist solved the disagreement by analyzing the respective data sources.

To compare the costs between different medication classes, we considered the least expensive drug of each class in each country (e.g., if loratadine was the least expensive OAH in a certain country, we assumed that the costs of being treated with OAH in that country corresponded to those of loratadine). We presented our results for one month of treatment, assuming full adherence to medication (i.e., that the medication was used every day of the month).

## 3 Results

We received 67 valid responses corresponding to a 20.0% response rate. The experts were from 51 countries, and we were able to obtain responses from 60.0% of the countries with ARIA experts (*N* = 85). The continent with the largest number of represented countries was Europe (*N* = 23), followed by Asia (*N* = 16), America (*N* = 9), Africa (*N* = 2), and Oceania (*N* = 1). For Africa, we obtained responses from 18.2% of the countries having ARIA experts; for Europe, Asia, and America, this percentage ranged between 63.9% and 69.2%.

By comparing drug classes, OAH was the cheapest drug class in most countries (36 countries), followed by INCS (12 countries) and INAH (4 countries). INAH+INCS was never identified as the cheapest drug class.

### 3.1 Intranasal Corticosteroids

INCS were available in all 51 countries. Among INCS, the most widely available were fluticasone propionate and mometasone furoate (48 countries). Those less widely available were dexamethasone (9 countries) and flunisolide (8 countries).

**FIGURE 1:**
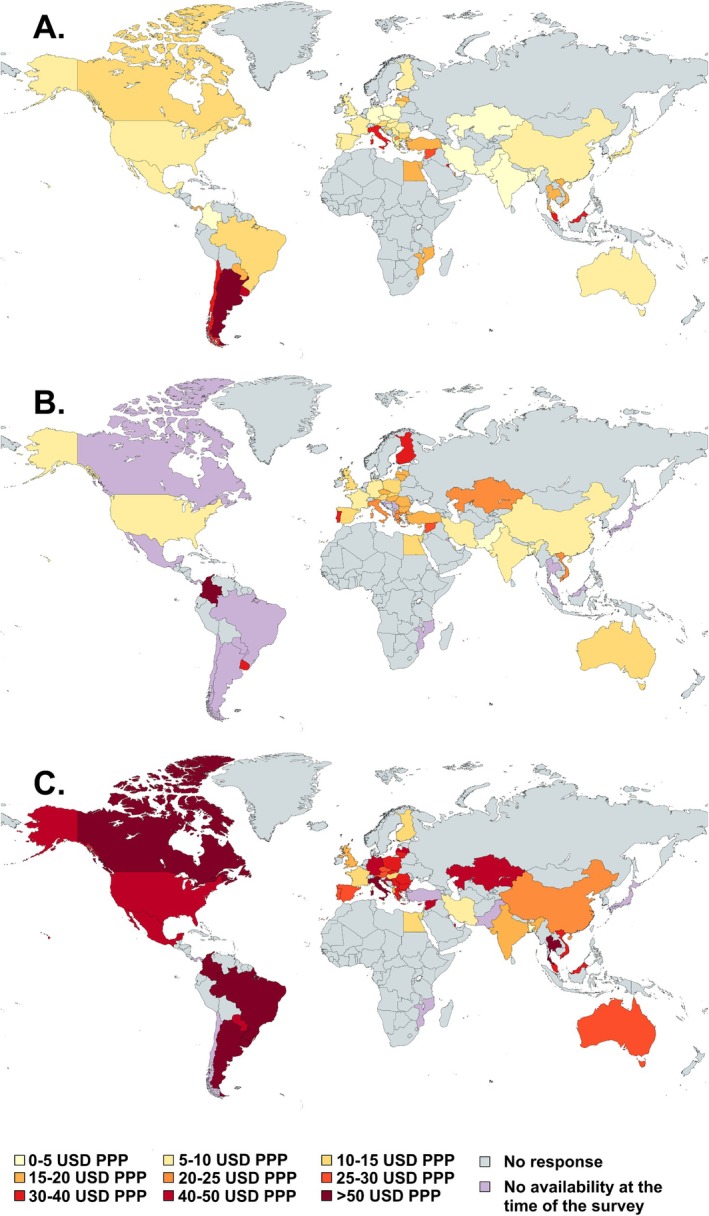
Monthly costs associated with the use of intranasal corticosteroids (A), intranasal antihistamines (B), or fixed combinations of intranasal antihistamines + corticosteroids (C). Monthly costs assume full adherence to medication use and the choice of the least expensive medication within each class. Data of 2024.

The lowest cost of INCS (assuming full adherence) was found in Israel, with a monthly cost per patient of 0.28 USD PPP. By contrast, the highest costs were reported at 57.67 USD PPP in Argentina (Figure 1A).

**FIGURE 2:**
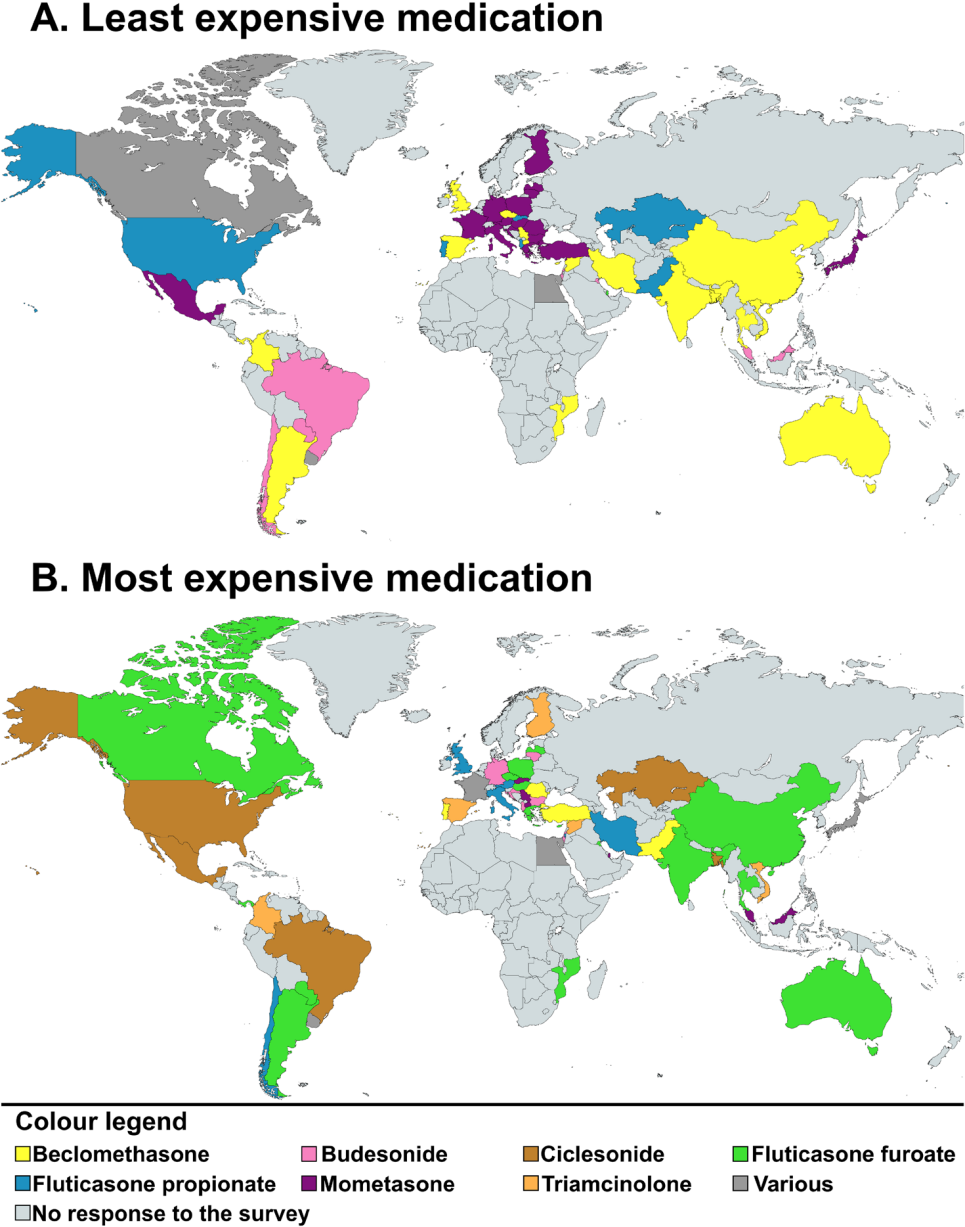
Least expensive (A) and most expensive (B) intranasal corticosteroid in each country. The colour of each country indicates the least expensive and the most expensive intranasal corticosteroid as of 2024.

Beclomethasone (*N* = 18) and mometasone furoate (*N* = 17) were identified as the cheapest medications in the largest number of countries, followed by fluticasone propionate (*N* = 7) and budesonide (*N* = 6) (Figure 2). Ciclesonide and triamcinolone were never identified as the cheapest drugs.

### 3.2 Intranasal Antihistamines

INAH were available in 35 countries (68.6%), with the most widely available being azelastine 0.10% (30 countries). All other INAH were reported to be available in less than half of the countries.

The lowest monthly costs of INAH were found in Bangladesh (1.5 USD PPP), whereas the highest were reported in Colombia (71.37 USD PPP) (Figure 1B).

Azelastine 0.10% was identified as the cheapest medication in the largest number of countries (*N* = 21).

### 3.3 Intranasal Antihistamines + Intranasal Corticosteroids

INAH+INCS were available in 45 countries (88.2%). Among INAH+INCS, the most widely available was azelastine + fluticasone (43 countries), which was the cheapest combination in 33 countries.

The lowest cost of INAH+INCS was found in Bangladesh (monthly cost per patient: 2.7 USD PPP); the highest cost was found in Argentina (100.75 USD PPP) (Figure 1C).

### 3.4 Oral Antihistamines

OAH were available in all 51 countries. Among OAH, the most widely available were cetirizine (51 countries) and loratadine (51 countries). On the other hand, ebastine was the less widely available (27 countries).

**FIGURE 3:**
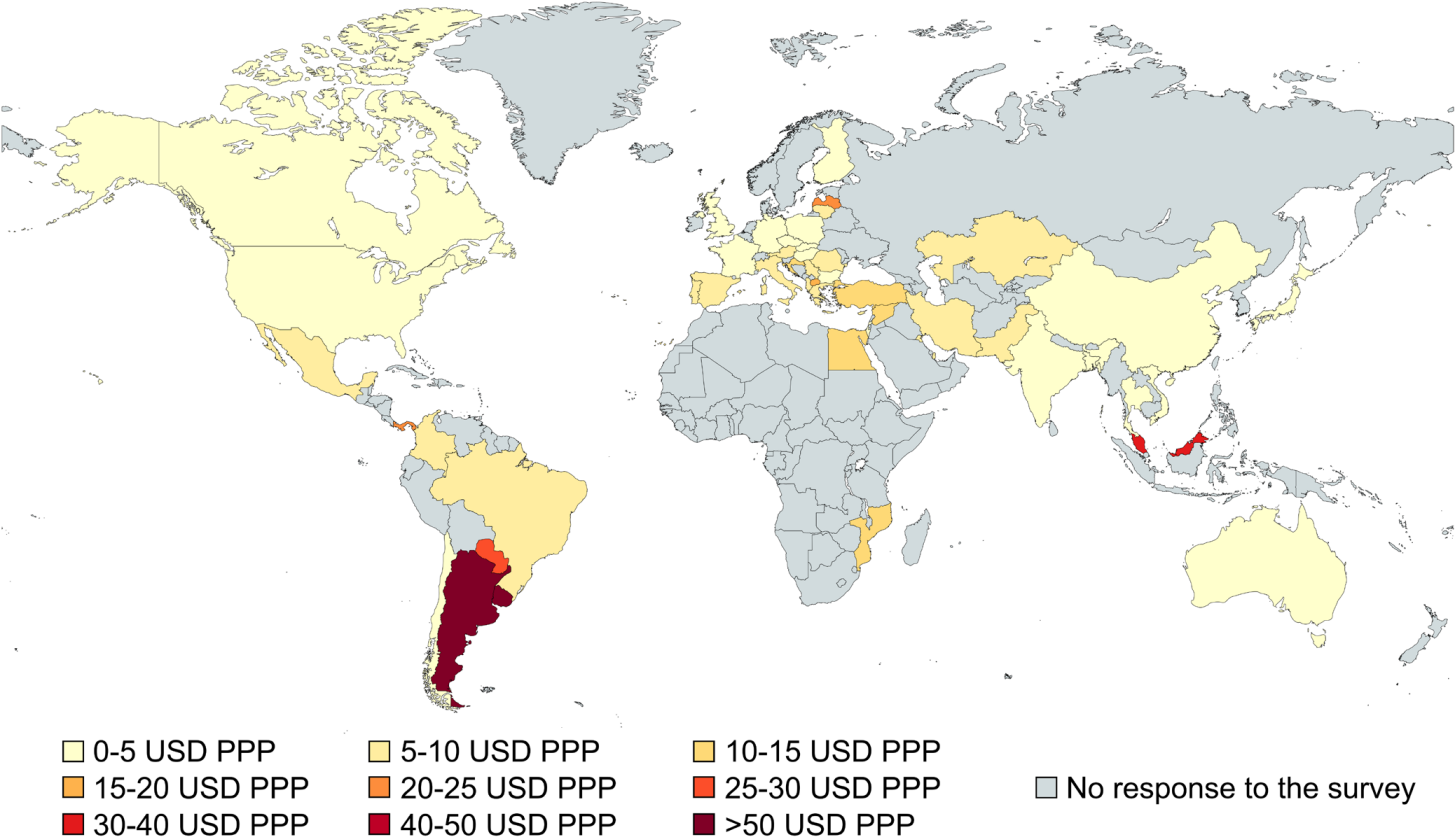
Monthly costs associated with the use of oral antihistamines. Monthly costs assume full adherence to medication use and the choice of the least expensive medication within each class. Data of 2024.

The lowest cost of OAH (assuming full adherence) was found in Hong Kong, with a monthly cost per patient of 0.39 USD PPP (Figure 3). By contrast, the highest costs were reported at 61.94 USD PPP in Argentina (Figure 3).

**FIGURE 4:**
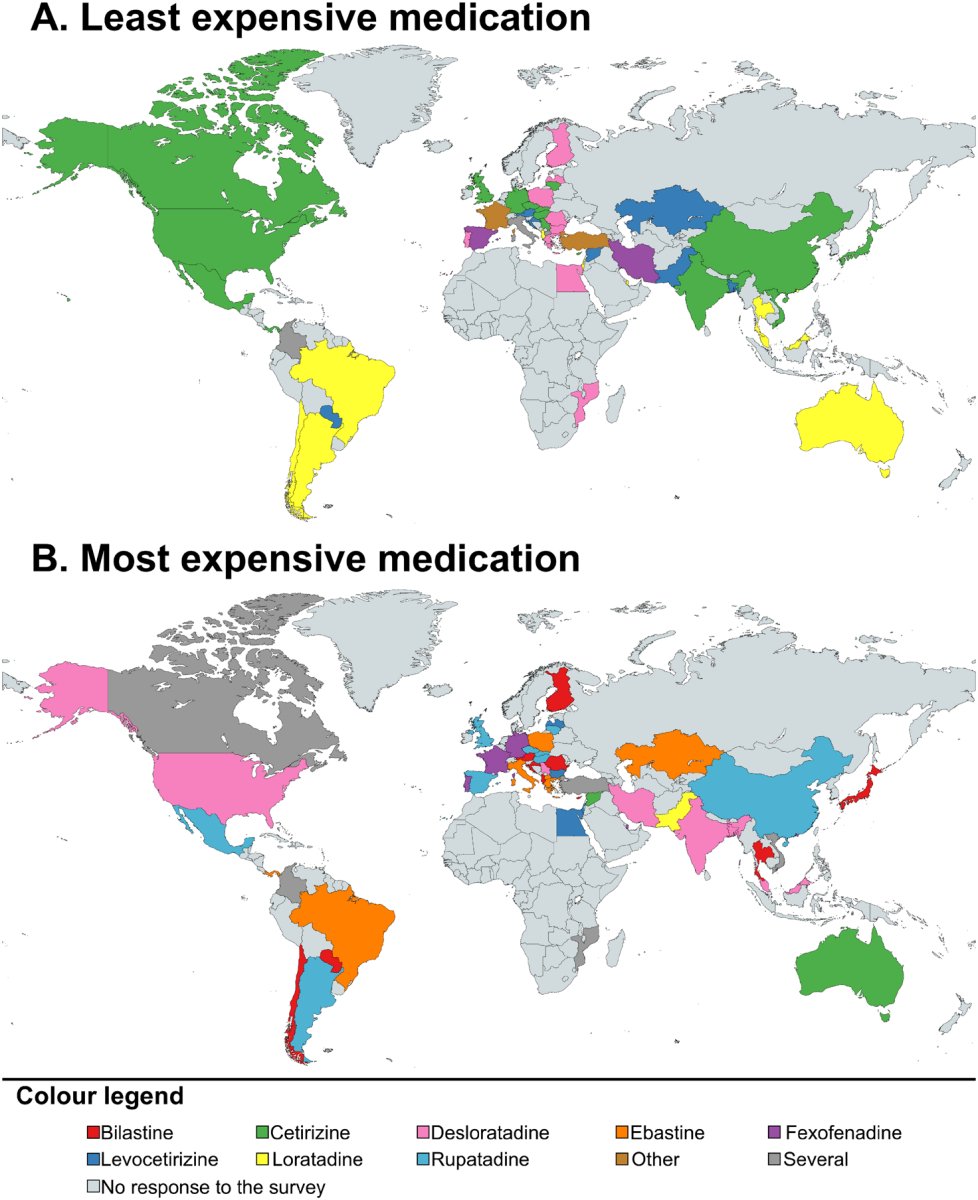
Least expensive (A) and most expensive (B) oral antihistamine in each country. The colour of each country indicates the least expensive and the most expensive oral antihistamine as of 2024.

Cetirizine was identified as the cheapest medication in the largest number of countries (*N* = 18), followed by desloratadine (*N* = 10) and loratadine (*N* = 10) (Figure 4). Bilastine was never identified as the cheapest drug.

## 4 Discussion

In this study, we surveyed ARIA experts and observed that INCS and OAH were the most widely available medication classes, with at least one medication being available in all assessed countries. In addition, in most countries, OAH are the cheapest medications. By contrast, INAH+INCS are most often the most expensive medications. However, this does not necessarily mean that INCS and INAH+INCS are not cost‐effective: a recent systematic review has reported that INCS and INAH+INCS are more effective than OAH [2], and it is possible that the society is willing to pay for the additional benefits resulting from the use of intranasal medications (rendering them cost‐effective). Furthermore, we assumed full adherence to the treatment, but this very rarely occurs [15, 16]. Patients can often adequately control their symptoms even if not fully complying with daily treatment. This introduces important variations in the actual expenses that patients have to incur for rhinitis medications. While this variability is influenced by the severity of the disease, it is also important to note that the capacity of achieving adequate control on an as‐needed basis may also vary according to the medication being used [15].

Overall, the lowest medication costs adjusted for PPP were found in Asia. In contrast, the highest costs tended to be in South America. While the reported values partly reflect differences in purchase power of the countries being compared, the same trend was observed when costs were expressed in USD not adjusted for purchase power. For INCS, the lowest costs were reported in Israel (0.3 USD), Bangladesh (1.3 USD), and India (2.5 USD), while the highest were reported in Argentina (26.5 USD) and Uruguay (30.7 USD). For OAH, the lowest costs were reported in Bangladesh (0.1 USD) and India (0.2 USD), and the highest in Argentina (28.4 USD) and Uruguay (35.7 USD). Of note, even within the same region, large across‐country variability in medication expenses was often observed. Importantly, there is also substantial across‐country variability in terms of reimbursement or coverage of medication costs: that is, there are some countries where patients (all or a subset) have access to reduced medications at a reduced price (examples based on the comments from ARIA experts are found in Supplementary Figure 1). Moreover, there are differences between regions in some countries. As an example, INCS are reimbursed in some but not most Italian regions, explaining differences in medication sale patterns [17].

To prescribe the best drug for each patient, it is important not only to consider the efficacy and safety of the medications but also other aspects such as acceptability and affordability. Considering that AR is a chronic disease, patients can have expenses through long periods of their lives with these medications. As suggested by studies in other chronic diseases, medication costs appear to have an impact on treatment adherence [18, 19, 20, 21, 22]. Options that can mitigate costs with AR medications include (i) prescribing the least expensive medications within each class that do not result in efficacy loss, (ii) prescribing generic medications when available, and (iii) adopting an as‐needed treatment strategy when adequate [23]. The ARIA‐EAACI 2024–2025 guidelines will support these options by providing evidence and recommendations on (i) different individual medications within the same class, and (ii) as‐needed versus chronic treatment strategies. In addition, the ARIA‐EAACI 2024–2025 guidelines provide indication on the medications available in the World Health Organization List of Essential Medicines. Both OAH and INCS—the two most available classes—have medications in that list, namely cetirizine, fexofenadine, loratadine (OAH), and budesonide (INCS) [24].

This study has some limitations. First, the data were self‐reported by experts in each country, and, in several countries, there was no possibility of verifying the reliability of the information using official sources. In addition, we observed a low response rate, with only one‐fifth of the ARIA experts having responded to the survey. Nevertheless, these experts represented 60% of the countries with ARIA members. However, it is important to note that the frequency of countries with experts answering the survey displayed geographic heterogeneity. As a result, there was underrepresentation in some regions, particularly in Africa, with only two responses from African countries (only one from Sub‐Saharan Africa) out of eleven with ARIA experts. In some African countries, pharmacists often stock the drugs that are prescribed or requested by patients. If there is no demand (due to low awareness of the benefits or low ability to pay), then pharmacists do not stock, resulting in an availability problem as well as a lack of competition that can keep prices high [25, 26]. Another limitation is that, for prescribed drugs, we did not collect information on the amount that was covered *vis‐à‐vis* that paid out of pocket by the patients. Finally, this study only aimed to examine the drug costs, so it does not provide information on the cost‐effectiveness of treatments. However, the data that we have collected can be used in future cost‐effectiveness studies.

This study also has important strengths. First, we received responses from all the continents with permanent inhabitants, allowing a global view of the costs across the world. Furthermore, while this study is based on the data reported by healthcare professionals, we surveyed local ARIA experts to ensure greater accuracy. Finally, despite the data having been obtained in each local currency, we presented results in USD PPP, enabling the comparison to take into account differences across countries in purchasing power.

In terms of implications, this study is directly informing the ARIA‐EAACI 2024–2025 guidelines. In particular, as these guidelines follow the GRADE methodology, the development of recommendations implies gathering evidence for twelve different criteria, namely those of the Evidence‐to‐Decision framework. Two of these criteria concern the costs (“resources required”) and cost‐effectiveness of interventions. The logic for this consideration is grounded on their implications: as an example, high costs may be a barrier to an adequate use of a medication—if patients cannot afford a treatment, their adherence may be suboptimal, which may reflect in an inadequate control of their symptoms. The results presented in this study may help health professionals choose the most effective medication (e.g., when choosing between different INCS or OAH) after considering the options available and affordable for the patient in the respective country. In addition, the results of this study support tailoring implementation considerations for low‐ and middle‐income countries. Of note, the ARIA‐EAACI 2024–2025 guidelines concern exclusively the pharmacological treatment of AR, as there are other EAACI guidelines being conducted on allergen immunotherapy (AIT) for AR. Moreover, AIT costs are a complex topic, as there are differences between AIT forms for reimbursement within and between countries (with changes in reimbursement policies being expected for the near future). As a result, this survey only evaluated the costs and availability of AR pharmacological treatments and did not inquire about AIT. Considering (i) the relevance of AIT for AR treatment and (ii) the importance of future economic evaluation studies comparing AIT versus pharmacological treatment, future surveys may replicate the methodology of this study to evaluate the costs and reimbursement of AIT across different countries and inform the EAACI guidelines on AIT.

In conclusion, we conducted a survey of ARIA experts to study the costs and availability of AR pharmacological treatments across the world. We observed that OAH were the most commonly available and, in most countries, the least expensive medication class. Regarding intranasal medications, INCS tended to be less expensive than INAH or INAH+INCS. Within each class, the cheapest medication varied across countries. The results of this study will inform the ARIA‐EAACI 2024–2025 guidelines.

## Author Contributions

M.C.U. Data analysis and manuscript writing. R.J.V. Study planning, methodology, and critical review and editing of the manuscript. YKX: Data collection, critical review, and editing of the manuscript. SGM: Methodology, and critical review and editing of the manuscript AB: Study management, and critical review and editing of the manuscript BA, MAA, MAAN, EAC, S.M.‐A., JA, KSB, E.C.B., M.C.‐G., L.R.C., E.C., L.C., D.C., G.C., D.K.C., I.C., A.A.C., D.D., B.G., M.A.G., I.G., T.H., E.H., W.H., M.H., T.I., ZI, JCI, FWSK, HK, MK, VK, YL, DELL, BMa, P.A.M., M.R.M., GMD, AM, FM, BMi, YM, MN, REOH, CPa, PP, NGP, JMFP, NPT, CPs, DP, MSRR, JR, MVR, MR, HS, PT, VTS, AV, LdlV, M.T.V., DW, PW, SW, FJPZ, LK, TZ: Data collection, and critical review and editing of the manuscript. A.M.P., JAF: Study planning, methodology, and critical review and editing of the manuscript JB: Study management, study planning, methodology, and critical review and editing of the manuscript. BSP: Study planning, methodology, data analysis, and manuscript writing.

## Funding

The work of this study has been funded within the context of the Allergic Rhinitis and its Impact on Asthma (ARIA) group.

## Conflicts of Interest

J. Bousquet reports personal fees from Cipla, Menarini, Mylan, Novartis, Purina, Sanofi‐Aventis, Teva, Noucor, other from KYomed‐Innov, other from Mask‐air‐SAS, outside the submitted work T. Iinuma reports grants from Sanofi, grants from Mochida, outside the submitted work. P. Tantilipikorn reports personal fees from ALK, personal fees from Abbott, personal fees from Viatris, personal fees from Menerini outside the submitted work. M. Kupczyk reports personal fees from Adamed, personal fees from Astra Zeneca, personal fees from Almiral, personal fees from Berlin Chemie Menarini, personal fees from Aurovitas, personal fees from Chiesi, personal fees from GSK, personal fees from Novartis, personal fees from Teva, personal fees from Stada, personal fees from Glenmark, personal fees from HVD, personal fees from Zentiva, personal fees from EMMA, personal fees from HAL Allergy, personal fees from Sunpharm, personal fees from Sanofi, outside the submitted work. D. Larenas Linnemann reports personal fees from ALK, Armstrong, Astrazeneca national and global, Chiesi, GSK national and global, Megalabs Ecuador, Naos, Novartis, Pfizer, Sanofi, Siegfried, Syneos Health, grants from Abbvie, Lilly, Sanofi, Astrazeneca, Pfizer, Novartis, GSK, Chiesi, Biopharma, outside the submitted work; and Editor in chief of Immune System (Karger)Ðember of asthma committee ACAAIÐubgroup chair of allergen immunotherapy Practice parameter update JTF AAAAI/ACAAI 2024Ðember of allergen immunotherapy committee AAAAIÜhair of allergen immunotherapy committee CMICAÐember of allergic asthma task force EAACI. JC. Ivancevich reports personal fees from Laboratorios Casasco Argentina, personal fees from World Allergy Organization, personal fees from Global Asthma Association (Interasma) outside the submitted work. N. Papadopoulos reports personal fees from Nestlé Nutrition Institute, personal fees from Abbott Nutrition, grants from Numil Hellas SA, grants from VIANEX, personal fees from GSK, personal fees from HAL Allergy Holding B.V, personal fees from Menarini International Operations Luxembourg SA, personal fees from Regeneron Pharmaceuticals Inc., personal fees from Berlin—Chemie AG, personal fees from DBV Technologies SA, grants from Vibrant America, personal fees from Hyproca Nutrition USA INC, personal fees from Danone Trading Medical B.V., personal fees from Med Maps srl, outside the submitted work. L. Cecchi reports personal fees from Astra Zeneca, personal fees from Menarini, personal fees from Sanofi, personal fees from GSK, personal fees from Firma, personal fees from Thermofisher, personal fees from Novartis, personal fees from Chiesi, outside the submitted work. J. Romantowski reports personal fees from GSK, personal fees from Sanofi, personal fees from AstraZeneca outside the submitted work. T. Zuberbier reports honoraria for lectures from Amgen, AstraZeneca, AbbVie, ALK ‐Abelló, Almirall, Astellas, Bayer Health Care, Bencard, Berlin Chemie, FAES Farma, HAL Allergie GmbH, Henkel, Kryolan, Leti, L'Oreal, Meda, Menarini, Merck Sharp & Dohme, Novartis, Nuocor, Pfizer, Sanofi, Stallergenes, Takeda, Teva, UCB, and Uriach; Fees for industry consulting were received from Abivax, Almirall, Bluprint, Celldex, Celltrion, Novartis, and Sanofi; in addition he declares non‐paid organizational affiliations: Committee member, “Allergic Rhinitis and its Impact on Asthma” (ARIA), Member of the Board, German Society for Allergy and Clinical Immunology (DGAKI), Head, European Centre for Allergy Research Foundation (ECARF), President, Global Allergy and Asthma Excellence Network (GA^2^LEN), and Member, Committee on Allergy Diagnosis and Molecular Allergology, World Allergy Organization (WAO). S. Williams reports: like many people, she has allergic rhinitis with her own personal preference for treatment, and this therefore required reflexivity when reviewing the research. As a result, she does not believe this has affected her judgment; in fact, it has informed us what she will do in the future. P. Werminghaus reports personal fees from Astrazeneca, personal fees from Allergy Therapeutic, personal fees from GSK, personal fees from Sanofi, personal fees from Stallergenes, personal fees from Celltrion outside the submitted work. A. Cruz reports grants, personal fees, and non‐financial support from AstraZeneca, personal fees from Chiesi, personal fees from GSK, personal fees from Farmoquimica, personal fees from Glennmark, grants and personal fees from Sanofi, personal fees from Sunvou outside the submitted work. D. Wallace reports that she was 2nd author on the JTFPP “Rhinitis 2020: A Practice Parameter Update”. However, Dr. Wallace does not feel that this has impacted her authorship role in the development of this manuscript. H. Kraxner reports speaker's fees and congress support from Sanofi, Viatris, Berlin‐Chemie, Ewopharma, and AstraZeneca; Advisory Board memberships of Sanofi, AstraZeneca, and Berlin‐Chemie. The other authors have nothing to disclose, outside the submitted work.

## Supporting information


**Supplementary Figure 1** Examples provided by ARIA experts of rhinitis medication costs being partly or fully covered by the health system.

## Data Availability

The data that support the findings of this study are available from the corresponding author upon reasonable request.
