# Supplementary material for: Availability and Costs of Allergic Rhinitis Treatments Across the World: A Survey of ARIA Experts
Source: Allergy. 2026 Apr 18;81(6):2069–78. doi: 10.1111/all.70340 (PMC13256277; doi:10.1111/all.70340)
Supplement: Supplementary file 1 — Supplementary Figure 1 Examples provided by ARIA experts of rhinitis medication costs being partly or fully covered by the health system. [file ALL-81-2069-s001.doc]

**Supplementary Figure 1. Examples provided by ARIA experts of rhinitis medication costs being partly or fully covered by the health system**

**
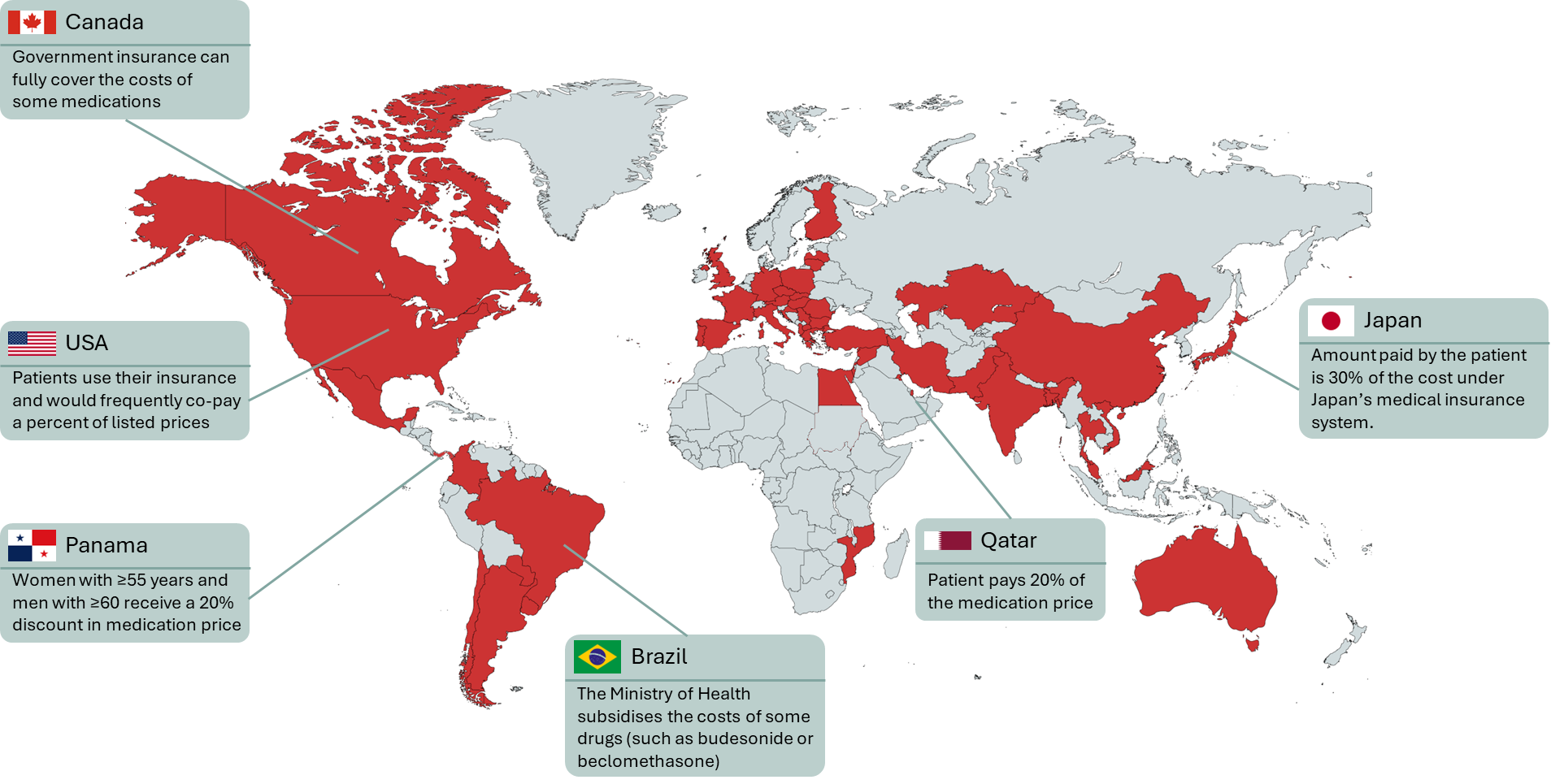
**
